# Ficolin-1 in pediatric *Plasmodium falciparum* malaria and its possible role in parasite clearance and anemia

**DOI:** 10.1128/iai.00194-25

**Published:** 2025-05-27

**Authors:** Di Zheng, Natalie Ferrington, Dilini Rathnayake, Wina Hasang, Agersew Alemu, Visopo Harawa, Amalia Karahalios, Phoebe Fitzpatrick, Evelyne Gout, Nicole M. Thielens, Karl Seydel, Terrie E. Taylor, Wilson Mandala, Stephen J. Rogerson, Elizabeth H. Aitken, Louise M. Randall

**Affiliations:** 1 Department of Medicine, Peter Doherty Institute of Infection and Immunity, University of Melbourne 2281, Melbourne, Victoria, Australia; 2 Department of Infectious Diseases, Peter Doherty Institute of Infection and Immunity, University of Melbourne 2281, Melbourne, Victoria, Australia; 3 Biomedical Sciences Department, College of Medicine, University of Malawi 37610, Blantyre, Malawi; 4 Malawi-Liverpool-Wellcome Trust Clinical Research Programme 560808, Blantyre, Malawi; 5 Blantyre Malaria Project, Kamuzu University of Health Sciences 37610, Blantyre, Malawi; 6 Centre for Epidemiology and Biostatistics, School of Population and Global Health, The University of Melbourne 50066, Melbourne, Victoria, Australia; 7 Methods and Implementation Support for Clinical Health (MISCH) Research Hub, Faculty of Medicine, Dentistry and Health Sciences, University of Melbourne 85084 , Melbourne, Victoria, Australia; 8 Univ. Grenoble Alpes, CEA, CNRS, IBS 27015, Grenoble, France; 9 College of Osteopathic Medicine, Michigan State University 43977 , East Lansing, Michigan, USA; 10 Academy of Medical Sciences, Malawi University of Science and Technology 542087, Thyolo, Malawi; 11 Department of Microbiology & Immunology, Peter Doherty Institute of Infection and Immunity, The University of Melbourne 2281, Melbourne, Victoria, Australia; 12 Population Health and Immunity Division, The Walter and Eliza Hall Institute of Medical Research 5388, Melbourne, Victoria, Australia; University of California Davis, Davis, California, USA

**Keywords:** ficolin, *Plasmodium falciparum*, severe malaria, Malawi

## Abstract

*Plasmodium falciparum* malaria causes significant disease, especially in young children. A successful immune response to *P. falciparum* is a major determinant of clinical outcome. The ficolins are a family of lectins that act as pattern recognition molecules and can activate the lectin complement pathway and may promote inflammation and facilitate opsonization and lysis of pathogens. Here, we have investigated the potential roles of ficolin-1 and ficolin-2 in the context of *P. falciparum* infection. We measured ficolin-1 and ficolin-2 concentrations in plasma from Malawian children presenting with uncomplicated or severe malaria or healthy controls (HCs) by ELISA. Using flow cytometry, we assessed whether ficolin-1 could bind to infected red blood cells (iRBCs) and whether it binds sialic acid on the iRBCs. Ficolin-1 and ficolin-2 plasma levels were measured in children from all clinical groups. Compared to HCs (reference), Ficolin-1 concentrations in plasma were higher in children with uncomplicated (geometric mean ratio: 1.88; 95% confidence interval [CI]: 1.25–2.82) and severe malaria (1.65; 95% CI: 1.10–2.46). Ficolin-1 levels were positively associated with peripheral blood monocyte (1.30; 1.02–1.67) and neutrophil counts (1.06; 1.00–1.13). Ficolin-2 was not associated with malaria. Hemoglobin levels were negatively associated with ficolin-1 (−0.38; −0.68 to –0.09) and ficolin-2 (−0.36; −0.68 to –0.04). Ficolin-1 bound more to iRBCs compared to uninfected RBCs, and binding was reduced in a ficolin-1 mutant that did not bind to sialic acid. These results highlight a largely overlooked role for ficolin-1 in the immune response to *P. falciparum* infection and point to a potential role for lectins contributing to parasite clearance and anaemia.

## INTRODUCTION

Malaria, caused by infection with *Plasmodium* spp. parasites, is a global health burden. Of the six species which cause disease in humans, *Plasmodium falciparum* causes the most severe disease and is estimated to be responsible for over 600,000 deaths each year, mostly children under the age of 5 years (1, 2). Antibody-mediated immunity is important for protection from malaria (3); however, the role of other serological components is less clear.

Ficolins are a family of lectin pattern recognition molecules which, when bound to a pathogen in a complex with mannose-binding lectin-associated serine proteases (MASPs), can elicit clearance by initiating the lectin complement pathway, promoting inflammation, phagocytosis, and/or pathogen lysis. Humans have three ficolins, ficolin-1, ficolin-2, and ficolin-3 (also referred to as M-ficolin, L-ficolin, and H-ficolin, respectively). Ficolin-1 is synthesized by monocytes, primarily in the bone marrow, as well as neutrophils and type II alveolar epithelial cells in the lung, while ficolin-2 is mainly synthesized by hepatocytes in the liver and ficolin-3 by the epithelial cells and hepatocytes in the liver and type II alveolar epithelial cells in the lung (4–7). The three ficolins have a common binding specificity for acetylated compounds but also recognize various glycan epitopes with different binding profiles. Ficolin-1 is known to recognize sialylated glycans, and ficolin-2 recognizes sulfated saccharides (8). These three ficolins have also been shown to bind to a variety of pathogen-associated molecular patterns, or PAMPS, including bacterial peptidoglyan, lipopolysaccharide, and teichoic acids of some bacterial envelopes, as well as beta-glucan in the cell walls of fungi (reviewed in reference 9).

In addition to evidence of their involvement in bacterial and fungal infection, ficolins may play a role in the immune responses to protozoa. Ficolin-1 and ficolin-3 can bind *Leishmania infantum* (10), ficolin-1, ficolin-2, and ficolin-3 can bind to *L. braziliensis* (11), and the parasite *Trypanosoma cruzi* is able to partially inhibit activation of the complement pathway by ficolin-2 by expressing a surface protein which binds the collagenous portion of the ficolin (12). Ficolin binding to *Plasmodium* spp. has not been previously assessed. There is a paucity of data on ficolin levels and how they are associated with infections (13, 14). However, one paper highlights a possible role for these lectins in *Plasmodium* spp. infections, showing ficolin-2 levels are associated with severe malaria (14).

To further understand whether ficolins play any role in the human immune response to malaria, we determined whether ficolin-1 and ficolin-2 concentrations varied with malaria disease and disease severity in plasma samples collected from children residing in a malaria endemic area. We then used *in vitro* assays to investigate binding of ficolin-1 to the infected red blood cells (iRBCs) and uninfected RBCs.

## MATERIALS AND METHODS

### Cohort

Children presenting with uncomplicated malaria (UM) or cerebral malaria (CM) were recruited at Queen Elizabeth Central Hospital, and HCs were recruited from the Ndirande Health Centre in Blantyre (which was offering an immunization program) (15), Malawi, from January 2016 to June 2017. HCs were children who had no *Plasmodium* spp. parasites detected on thick blood film examination and no known infection at the time of recruitment. UM was defined in children with fever and *P. falciparum* parasites on thick blood film but without signs or symptoms of severe malaria. CM was defined in children with fever, *P. falciparum* parasites on blood film, and a Blantyre score (16) of two or less both at admission and 4 h later, after ruling out other potential causes of coma such as hypoglycemia. Blood samples used for measurement of ficolin-1 and ficolin-2 were collected at presentation in sodium heparin anti-coagulated tubes. At presentation, age and sex were recorded, hemoglobin (Hb) and leukocyte counts (Coulter counters used were Beckman Coulter AcT5 Diff and Cap Piercing) were measured, and stained blood smears were made to assess parasitemia in individuals with severe malaria (17).

### Cell culture

*P. falciparum* parasite strains used included CS2, which expresses the *P. falciparum* erythrocyte membrane protein 1 (PfEMP1) VAR2CSA on the surface of the iRBCs, the CS2 parent line E8B which constitutively expresses a different PfEMP1 to CS2 (18) and a CS2 skeleton binding protein knock out (CS2SBP1KO), which does not export PfEMP1 to the surface of the iRBCs (19). All parasites were cultured in Roswell Park Memorial Institute-2 hydroxyethylpiperazine-N-2-ethanesulfonic acid medium with 0.2% wt/vol NaHCO_3_ supplemented with 0.5% Albumax II (Gibco). Blasticidin 3 µg/mL was added to the CS2SBP1KO strain (19). RBCs were supplied by the Australian Red Cross Blood Services. Cultures were maintained as previously described (20).

### Measurement of ficolin-1 and ficolin-2 in plasma

Concentrations of ficolin-1 and ficolin-2 were measured in plasma using sandwich ELISAs. The ficolin-1 ELISA was based on a previously published protocol (21). Briefly, each well of a 96-well plate (Maxisorp, Nunc) was coated with 1 µg/mL of mouse anti-human ficolin-1 antibody 7G1 (Hycult Biotech) in phosphate-buffered saline (PBS) and incubated at 4°C overnight. The plate was washed with PBS-Tween (PBS-T, 0.05% vol/vol) and blocked with blocking buffer (1% bovine serum albumin [BSA] in PBS wt/vol) for 2 h at room temperature (RT). The plate was then washed before 100 µL/well of standards made of recombinant human ficolin-1 (R&D Systems) or unknown plasma diluted 1:5 in PBS were added to the plate in duplicate and left for 3 h at 37°C. After washing in PBS-T, 100 µL of biotinylated mouse anti-human ficolin-1 (0.4 µg/mL, Hycult Biotech) was added to each well for 1 h at RT. The plates were then washed before streptavidin-horseradish peroxidase (HRP) (MabTech) was added for 1 h at RT. Plates were washed again before adding substrate (3,3′, 5,5′ tetramethylbenzidine, BD OptEIA), allowing the color to develop and stopping the reaction with 1 M H_2_SO_4_. The optical density was read at a wavelength of 450 nm using an Omega BMG Labtech microplate reader, and ficolin-1 plasma concentrations were extrapolated from the standard curve.

To measure ficolin-2, each well of a 96-well plate (Maxisorp, Nunc) was coated with 100 µL of 0.58 µg/mL mouse anti-human ficolin-2 antibody mAb16 (Abcam ab112558) in PBS and incubated at 4°C overnight. The plate was washed with PBS-T and blocked with blocking buffer for 2 h at RT. The plate was then washed before 100 µL/well of standards made of recombinant human ficolin-2 (Abcam) or plasma diluted 1:200 or 1:600 in PBS were added to the plate in duplicate and left to incubate overnight at RT. After washing in PBS-T, 100 µL of 0.25 µg/mL mouse IgG2a anti-human ficolin-2 mAb19 (Abcam, ab56225) was added to each well for 1 h at RT. The plate was then washed again, and 100 µL/well of 0.25 µg/mL biotinylated goat anti-mouse IgG2a antibody (Abcam) was added to the plate and left to incubate at RT for 1 h. The plate was again washed, and Streptavidin-HRP and substrate OptEIA were added before the reaction was stopped, read, and ficolin-2 plasma concentrations were obtained as described above for ficolin-1.

### Ficolin-1 binding to iRBCs

Trophozoite stage iRBCs were enriched by gelatin flotation (22), washed, and resuspended at 0.2% hematocrit in 20 µg/mL of human recombinant ficolin-1 (R&D Systems) or ficolin-1 Y271F (8) in 0.1% Casein (Thermo Fisher Scientific) in PBS, and left at 37°C for 30 min. A total of 2 μg/mL mouse anti-human ficolin-1 (Hycult Biotech mAb7G1) was added, followed by 4 µg/mL of polyclonal goat anti-mouse Alexa Fluor 647 (Invitrogen, A-11029) and 25 µg /mL of dihydroethidium (DHE). RBCs were gated by forward and side scatter, iRBCs were identified by DHE staining, and the presence and intensity of ficolin-1 on iRBCs and uninfected RBCs were determined by median fluorescence intensity of Alexa Fluor-647. Experiments were performed in triplicate and repeated using parasites cultured in RBC from at least four different donors.

### Statistical analysis

To assess whether ficolin-1 and ficolin-2 were correlated, we used Spearman’s correlation. To investigate the association between clinical variables and ficolin-1 and ficolin-2 levels, we fitted separate linear regression models to estimate the association between the logarithm of ficolin-1 and ficolin-2 concentrations with the following clinical variables measured at enrollment: white blood cell count, monocyte count, neutrophil count, and peripheral blood parasitemia at enrollment (parasites/μL). The estimated coefficients were back transformed and represent the ratio of geometric means. We estimated the association between ficolin-1 and ficolin-2 concentrations (log scale) with Hb levels by fitting a linear regression model. The estimated coefficients represent a change in Hb levels for a ~2.71 unit change in ficolin-1 or ficolin-2. All analyses were adjusted for age (in months). The assumptions of the linear regression models (i.e., linearity, normality, and homoscedasticity) were checked by visual inspection.

## RESULTS

Clinical data were available for 324 individuals, but due to sample limitations, we measured ficolin-1 in 176 individuals (which included 47 HCs, 63 with UM, and 66 with CM) and ficolin-2 in 193 individuals (including 62 HCs, 66 with UM, and 65 with CM) (see Fig. S1). HCs were younger than those in the UM and CM groups (median age 24 [12, 60] months compared to 48 [25, 84] months in the UM and 48 [29, 69] months in the CM group), the sex distribution was similar in all three groups, and the Hb was highest in the HCs at 11.6 (1.7) g/dL, compared to the UM and CM groups (9.8 [1.9] and 8.3 [1.8] g/dL, respectively) (see Table 1 for clinical characteristics of the three groups). Ficolin-1 concentrations ranged from 0.02 to 1.63 µg/mL, and ficolin-2 concentrations ranged from 0.1 to 12.70 µg/mL. For 152 samples with measures of both ficolin-1 and ficolin-2, there was no evidence that ficolin-1 and ficolin-2 were correlated (Spearman’s *ρ* = −0.04; 95% confidence interval [CI]: −0.20 to 0.12, *P* = 0.612).

**TABLE 1:** Characteristics of the study participants by clinical group^*a*^^,*c*^

Characteristic	Healthy control	Uncomplicated malaria	Severe malaria
*N* ^*b*^	69	70	78
Sex, female, *n* (%)	35 (51) [68]	37 (53)	49 (51)
Age (months) at enrollment, median (IQR)	24 (12–60)	48 (25–84)	48 (29–69)
White blood cell count (×10^3^/μL), median (IQR)	7.8 (6.4–9.1) [65]	8.3 (6.2–10.6) [68]	9.1 (6.1–11.0) [57]
Monocyte count (×10^3^/μL), median (IQR)	0.51 (0.4–0.81) [63]	0.70 (0.48–1.02) [56]	0.79 (0.53–1.71) [52]
Neutrophil count (×10^3^/μL), median (IQR)	2.1 (1.8–2.7) [64]	4.6 (3.2–7.4) [55]	3.9 (2.7–6.2) [51]
Parasitemia (×10^3^/μL), median (IQR)^C^	-	-	2.97 (0.30–111.28) [77]
Hemoglobin (g/dL), mean (SD)	11.6 (1.7) [65]	9.8 (1.9) [68]	8.3 (1.8) [73]

^*a*^ Data are median (interquartile range) or mean (standard deviation). Abbreviations: IQR, interquartile range (25th and 75th percentiles).

^*b*^ The number of participants with available data is noted in square brackets when it differs to *N*.

^*c*^ Parasitemia was only available for individuals with severe malaria. “-” denotes not calculated.

Ficolin-1 concentrations were higher in children with UM (1.88; 95% CI: 1.25–2.82) and CM (1.65; 1.10–2.46) than in HCs (reference group) (Table 2). By contrast, ficolin-2 was not increased in either UM or CM compared to HCs (Table 3). When the distribution of the ficolin levels was plotted by group, there was no difference in concentrations of ficolin-2 or ficolin-1 between those with UM and CM.

**TABLE 2:** Association between the concentration of ficolin-1 (μg/mL) and selected characteristics after adjusting for age

			95% Confidence interval (CI)		
Variable	*n*	Geometric mean ratio^*a*^	Lower limit	Upper limit	*P* vale
Clinical group					
Healthy control	47	Ref			
Uncomplicated malaria	63	1.88	1.25	2.82	0.003
Severe malaria	66	1.65	1.10	2.46	0.016
White blood cell count (×10^3^/μL)	150	1.03	0.98	1.07	0.229
Monocytes (×10^3^/μL)	135	1.30	1.02	1.67	0.036
Neutrophil count (×10^3^/μL)	134	1.06	1.00	1.13	0.044
Parasite count (×10^3^/μL)	65^*b*^	1.00	1.00	1.00	0.637

^*a*^ Geometric mean ratio denotes the proportional increase/decrease in ficolin-1 (μg/mL) by having either types of malaria (uncomplicated or severe) or for each unit increase in the continuous variables. The geometric mean ratios (95% CIs) were estimated by fitting linear regression models adjusted for age (measured in months) and with the outcome, concentration of ficolin-1, on the log scale.

^*b*^ Only participants with severe malaria have parasite count data.

**TABLE 3:** Association between the concentration of ficolin-2 (μg/mL) and selected characteristics after adjusting for age

			95% Confidence interval (CI)		
Variable	*n*	Geometric mean ratio^*a*^	Lower limit	Upper limit	*P* value
Clinical group					
Healthy control	62	Ref			
Uncomplicated malaria	66	1.10	0.77	1.56	0.611
Severe malaria	65	1.29	0.90	1.83	0.161
White blood cell count (×10^3^/μL)	168	0.98	0.94	1.01	0.189
Monocytes (×10^3^/μL)	151	0.91	0.75	1.10	0.345
Neutrophil count (×10^3^/μL)	149	0.96	0.92	1.02	0.167
Parasite count (×10^3^/μL)	64^*b*^	1.00	1.00	1.00	0.949

^*a*^ Geometric mean ratio denotes the proportional increase/decrease in ficolin-2 (μg/mL) by having either types of malaria (uncomplicated or severe) or for each unit increase in the continuous variables. The geometric mean ratio (95% CIs) were estimated by fitting linear regression models adjusted for age (measured in months) and with the outcome, concentration of ficolin-2, on the log scale.

^*b*^ Only participants with severe malaria have parasite count data.

Because ficolin-1 is produced by leukocytes (23), we examined the association between concentrations of ficolin-1 and ficolin-2 and peripheral blood monocyte and neutrophil counts. Ficolin-1 concentration was positively associated with both peripheral blood monocyte and neutrophil counts (Table 2), but, as expected, ficolin-2 was not (Table 3). When we examined the relationship between ficolin-1 and the peripheral blood parasite density, there was no evidence of an association between ficolin-1 and number of parasites/μL of blood, in individuals with CM (1.00; 1.00–1.00) (Table 2). We did not have data for parasitemia for individuals with UM, and as such, no similar comparison could be conducted with this group. When we examined for possible association between ficolin-2 and parasitemia, none was found (Table 3).

We investigated the relationship between ficolin concentrations and Hb levels. Controlling for age, we saw a negative association between ficolin-1 and Hb levels (−0.38; −0.68 to –0.09), and a similar but weaker association was seen with ficolin-2 (−0.36; –0.68 to –0.04) (Table 4).

**TABLE 4:** Association between hemoglobin (g/dL) and logarithm of concentration of ficolin-1 and ficolin-2 (μg/mL)

			95% Confidence interval (CI)		
Variable	*n*	Coefficient^*a*^	Lower limit	Upper limit	*P* value
Ficolin-1 (log scale)	168	−0.38	−0.68	−0.09	0.011
Ficolin-2 (log scale)	183	−0.36	−0.68	−0.04	0.029

^*a*^ Coefficients (95% CI) were estimated from a linear regression adjusted for age (measured in months) and fitting ficolin-1 and ficolin-2 on the log scale.

**Fig 1:**
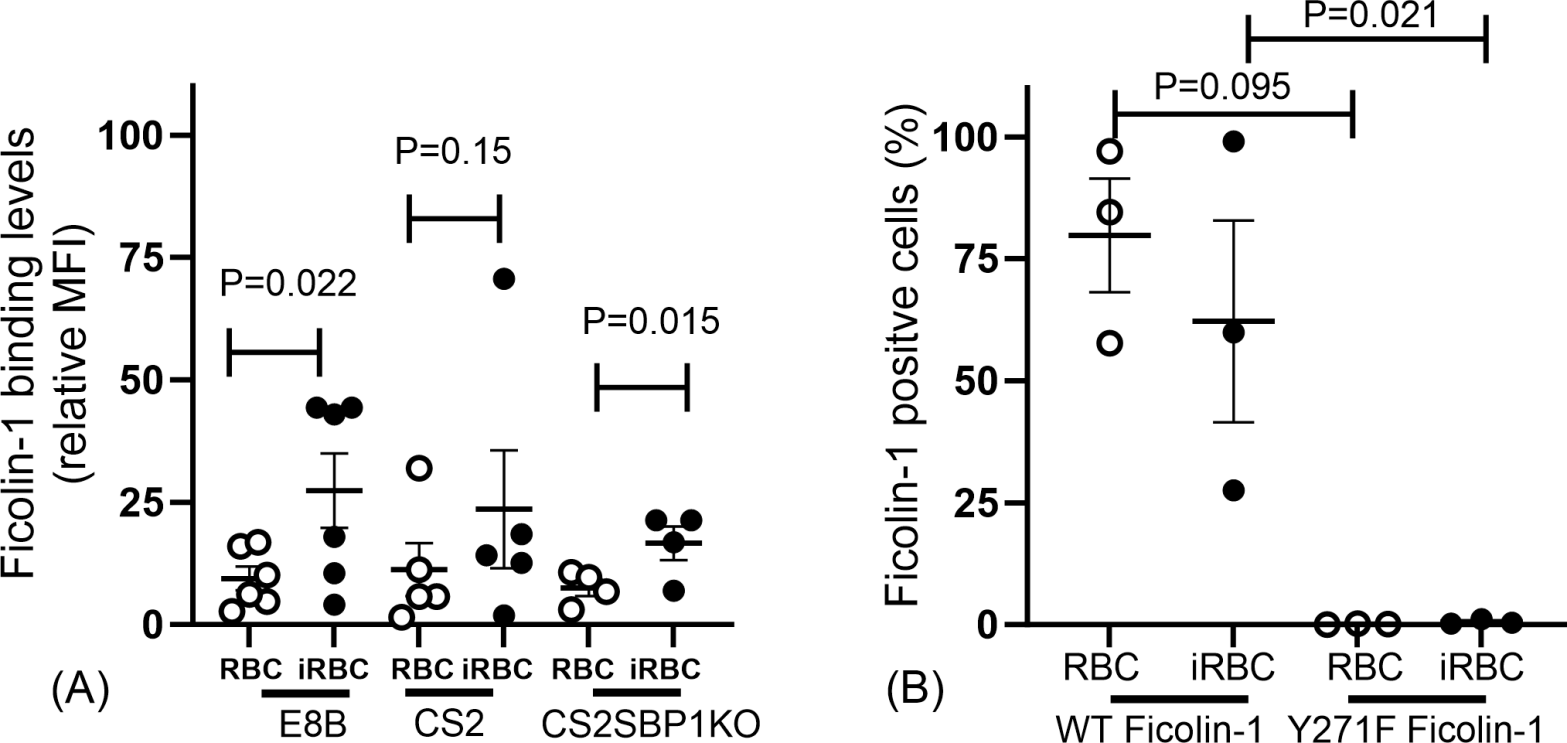
Ficolin-1 binding to *Plasmodium falciparum* infected red blood cells (iRBCs). (**A**) More ficolin-1 binds iRBC compared to uninfected RBCs. This was consistently seen for parasite isolates expressing different PfEMP1s (CS2 and E8B) and a mutant (CS2SBP1KO) which does not export PfEMP1 to the surface of the iRBCs. (**B**) The % of iRBCs (E8B strain) and RBCs which bound ficolin-1 was reduced when a mutant Y271F (which does not bind sialic acid-containing glycans) was used. Each data point is a mean of an individual experiment done in triplicate. Each experiment was repeated three to six times with different RBC donors. Bars are mean (SEM), means were compared using paired *t*-tests.

We examined whether ficolin-1 may play a role in the immune response to *P. falciparum* using *in vitro* models. We first studied whether ficolin-1 could bind to the surface of RBCs (Fig. 1). Ficolin-1 binding could be seen on both uninfected RBCs and iRBCs of most donors. This binding appeared greater for iRBCs, with a trend for increased binding to the CS2 parasite strain, which expresses the PfEMP1 VAR2CSA on the iRBC surface and E8B-ICAM which expresses a different PfEMP1 on its surface (*P* = 0.022). To determine whether the binding was dependent on PfEMP1 expression, we used SBP1-KO iRBC, which have decreased PfEMP1 surface expression. The SBP1-KO iRBC bound ficolin-1 more compared to the uninfected RBCs (*P* = 0.015), suggesting that ficolin-1 binding was not dependent on the presence of PfEMP1.

We used the ficolin-1 mutant (Y271F) whose mutation abolishes its binding to sialic acid-containing glycans (8). The ficolin-1 mutant did not bind to any iRBCs or uninfected RBCs, in contrast to the wild-type protein (Fig. 1B), suggesting that sialic acid is the primary ligand for ficolin-1 on both iRBCs and uninfected RBCs.

## DISCUSSION

The paper investigates the possible roles of ficolin-1 and ficolin-2 in *Plasmodium* spp. infections. We present evidence that individuals with clinical malarial illness have significantly higher concentrations of ficolin-1 compared to HCs. We also show that ficolin-1 binds to both infected and uninfected RBCs and evidence suggesting this binding is via sialic acid. Unlike ficolin-1, we did not see associations between ficolin-2 concentrations and any of the two clinical types of malaria studied.

The relationship between inflammation and ficolin-1 is largely unknown as few studies have measured ficolin-1 concentrations in the blood. This lack of data may be because it was thought that ficolin-1 was largely absent from plasma and instead localized to the surface of monocytes or in secretory granules of leukocytes (reviewed in reference 8). This is the first time that ficolin-1 concentrations have been measured in a cohort of individuals with malaria, and we saw that ficolin-1 was increased in those with disease compared to HCs.

It is likely that the increased concentrations of ficolin-1 in children with malaria in this cohort are due to increased numbers of leukocytes in those infected with *Plasmodium* spp. at different stages of the disease. Ficolin-1 is produced and secreted by both monocytes (24) and neutrophil subsets (4), and both leukocytes are increased with disease in *P. falciparum* infections (reviewed in references 25, 26). We also saw a positive correlation between monocyte and neutrophil counts and ficolin-1 levels in this cohort, supporting this hypothesis. Ficolin-1 levels in plasma have previously been positively associated with white blood cell (WBC) counts in individuals with autoimmune diseases (27) and children with cancer (28), but not in healthy individuals (29). In contrast, ficolin-1 has been shown to be decreased in patients with HIV (13), which could be linked to the decrease in WBC counts associated with HIV. It is also possible that ficolin-1 concentrations are raised due to increased secretion by stimulated cells. It is known that ficolin-1 is released along with neutrophil degranulation (4), which likely occurs in malaria (30). Circulating monocytes express significant levels of ficolin-1 mRNA (reviewed in reference 31), but whether or not stimulated monocytes secrete more ficolin-1 is not clear.

The concentrations of ficolin-1 in the cohort were comparable to those previously seen in diseased and healthy populations (28, 29, 32). It is worth noting that Munthe-Fog et al. hypothesized that ficolin-1 in measured plasma may be increased due to its release from monocytes in the presence of calcium chelators (29). The plasma from this study was collected using heparin (instead of a calcium chelator), and therefore, the measured values are likely to be reflective of those in circulation.

Sialic acid is a known ligand for ficolin-1 (8, 33), and the lack of observed binding of the ficolin-1 mutant Y271F, which is unable to bind sialylated glycans (8), suggests that sialic acid contributes to ficolin-1 binding on both the RBCs and iRBCs. Sialic acids are sugars that are found at the end of glycan chains on the surface of many cells. Ficolin-1 shows differential binding to different sialic acid-containing glycans, binding strongly to 9-O acetylated 2–6 linked sialic acid derivatives and sialic acid glycans with a 2–3 linkage (8). We do not know the sialic acid glycans to which ficolin-1 binds on iRBCs. As there is higher binding to the iRBCs compared to RBCs, we hypothesize that the sialic acids involved are associated with RBC modifications which occur (or are associated) with infection. This binding is probably to sialic acid of host (and not parasite) origin, as *P. falciparum* parasites have not been described to produce sialic acid-containing glycans (reviewed in reference 34). There was no evidence of involvement of the major parasite iRBCs surface antigen PfEMP1 in ficolin-1 binding, as ficolin-1 was able to bind iRBCs expressing different PfEMP1 variants as well as iRBCs which lack PfEMP1 surface expression.

It has been shown that sialic acid levels are increased 2- to 10-fold in mature iRBCs isolated from animal models of *Plasmodium knowlesi and Plasmodium berghei* (35, 36); however, it is unknown whether these sialic acids are exposed on the surface of the iRBCs (rather than in the parasitophorous vacuole membrane or elsewhere in the cell). Most of the sialic acid on the RBC surface is on the glycophorins, especially glycophorin A (reviewed in reference 37). There has been extensive research in the role of glycophorins as merozoite receptors (38), but little is known about the positioning and levels of glycophorin A in the iRBCs compared to the uninfected RBCs.

Another possible explanation for the observed preferential binding of ficolin-1 to the iRBCs is that there was preferential infection of younger RBCs by the parasite. Desialylation of RBC glycoproteins occurs as RBCs age (39), and if merozoites preferentially invade younger RBCs with more sialic acid on their surfaces, this could explain the results. However, unlike for *Plasmodium vivax*, an age preference for *P. falciparum* merozoites has not been shown, and it is widely regarded that they have no preference (40).

Binding of ficolin-1 to both the iRBCs and uninfected RBCs may contribute to their lysis, parasite clearance, and potentially anaemia. When ficolins bind a target, the associated MASP-2 enzyme is activated, resulting in the cleavage of complement proteins C4 and C2 and the activation of the lectin complement pathway (41), which can result in the deposition of opsonin C3 on the surface of the pathogen, production of inflammatory mediators such as C5 and cell lysis from the formation of the membrane attack complex. It is plausible that increased levels of ficolin-1 with infection are contributing to parasite clearance and even possibly to anaemia via cell lysis. In malaria, lysis of RBCs far exceeds that which would occur due to lysis of the parasite iRBC alone, and it is thought that the lysis of large numbers of uninfected RBCs contributes significantly to anaemia in infected individuals (42). As ficolin-1 was negatively associated with Hb concentration, we propose that increased levels of ficolin-1 with infection could contribute to malaria-related anaemia by increasing lysis of uninfected RBCs in addition to iRBCs. It should be noted that RBCs are partially protected against (43) complement-mediated lysis due to RBC protein CD59 inhibiting the formation of the membrane attack complex pore by C9 (43).

In addition to the activation of the complement pathway, the ability of ficolin-1 to bind iRBCs may contribute to monocyte activation by the parasites and inflammation. Secreted ficolin-1 can dock onto the monocyte surface via a G protein-coupled receptor 43 (GPCR43), where upon binding to a pathogen, it can activate the monocyte, resulting in increased IL-8 production (44). C-reactive protein is also able to bind ficolin-1 in this complex, and depending on the local pH, this binding exposes or hides the ficolin-1 pathogen binding site, resulting in up- or downregulation of IL-8 secretion (44).

Unlike with ficolin-1, we did not see a clear association between ficolin-2 concentrations and malaria. This was surprising as ficolin-2 concentrations in plasma have previously been shown to be increased with infectious diseases including dengue (45) and malaria (14). In Gabonese children, concentrations of ficolin-2 were higher at presentation in children with severe malaria than in those with uncomplicated disease. Our primary analysis did not involve comparing levels in children with CM and UM; however, the median levels of Ficolin-2 were very similar in both groups.

Unlike the Malawi cohort, the Gabon cohort included individuals with other clinical syndromes of severe disease who did not have CM, and the Malawi cohort was also half the size of that from Gabon. It is possible that differences in findings result from differences in syndromes included in the severe disease cohorts in the two studies. It is possible that our measures of ficolin-2 may have been influenced by the anti-coagulant we used, as ficolin-2 binds heparin, and it could have been complex with this compound in the ELISA. However, the range of concentrations measured in the Malawi cohort aligns well with previously published studies (14, 46), suggesting this is unlikely to be the explanation. It may be that the cohort in Malawi is genetically dissimilar to that in Gabon, as ficolin-2 levels are partly determined by host genetics, and the presence of a specific SNP in the ficolin-2 promoter has been associated with variations in ficolin-2 levels (47, 48). Interestingly, different haplotypes were not associated with disease severity in the Gabon cohort (14). Whether ficolin-2 levels vary with malaria severity needs to be investigated further.

Though there was no association between ficolin-2 and malaria, there was a negative association between ficolin-2 and Hb. Ficolins vary in their binding specificities (49), and we did not investigate whether ficolin-2 could bind RBCs or iRBCs. To our knowledge, a negative association between Hb and ficolin-2 has not previously been reported in any cohort, and whether ficolin-2 plays a role in anaemia is worth further investigation.

This study has several strengths. It utilizes a well-described cohort to investigate novel associations between ficolin and malaria. It then complements these observations with *in vitro* studies to examine experimentally possible roles and mechanisms of ficolin and disease. However, the study had several weaknesses. First, although clinical categories of disease are clearly described, there is missing data for parasitemia and leukocyte counts, which reduces the power of the study. Second, this work could have benefited from a larger sample size, which would have allowed us to dissect associations within clinical groups. In addition, the groups were not well matched in regard to age, so we needed to include this variable in our models. The role of ficolin in the activation of the complement pathway and RBC lysis could have been investigated by measurement of the deposition of complement proteins on the surface of the cells, and the identification of the host ligand sialic acid could have been confirmed further by the removal of sialic acid from the surface of the RBCs.

Using samples from a well-described cohort in Malawi, we show for the first time that ficolin-1 is increased with malaria and inflammation and is negatively associated with Hb. We show that ficolin-1 preferentially binds iRBCs but that it can also bind uninfected RBCs at lower levels. Use of a ficolin-1 mutant suggested that the ficolin-1 ligand on the iRBCs and uninfected RBCs was sialic acid, and use of mutant parasites suggested it was unlikely that the target was parasite protein PfEMP1. The increased binding of ficolin-1 to the iRBCs suggests altered levels of sialic acid on the surface of the iRBCs compared to uninfected RBCs. This has not been previously described and warrants further investigation. We also make the novel observation that ficolin-2 levels are negatively associated with Hb. However, the data in the paper suggest that concentrations of ficolin-2 are not higher in those with severe compared to uncomplicated malaria, which contrasts with a previously published study. Further studies using well-described cohorts are needed to confirm the associations between ficolin-2 and disease.

In conclusion, the data presented in this paper suggest for the first time that ficolin-1 may have an important role in malaria disease.

## SUPPLEMENTAL MATERIAL

Supplemental material iai.00194-25-s0001.docx Fig. S1 and S2.

ASM does not own the copyrights to Supplemental Material that may be linked to, or accessed through, an article. The authors have granted ASM a non-exclusive, world-wide license to publish the Supplemental Material files. Please contact the corresponding author directly for reuse.
