# Supplementary material for: Ficolin-1 in pediatric Plasmodium falciparum malaria and its possible role in parasite clearance and anemia
Source: Infect Immun. 2025 May 27;93(7):e00194-25. doi: 10.1128/iai.00194-25 (PMC12234430; doi:10.1128/iai.00194-25)
Supplement: Supplemental material — Fig. S1 and S2. [file iai.00194-25-s0001.docx]

**Supplementary Material**


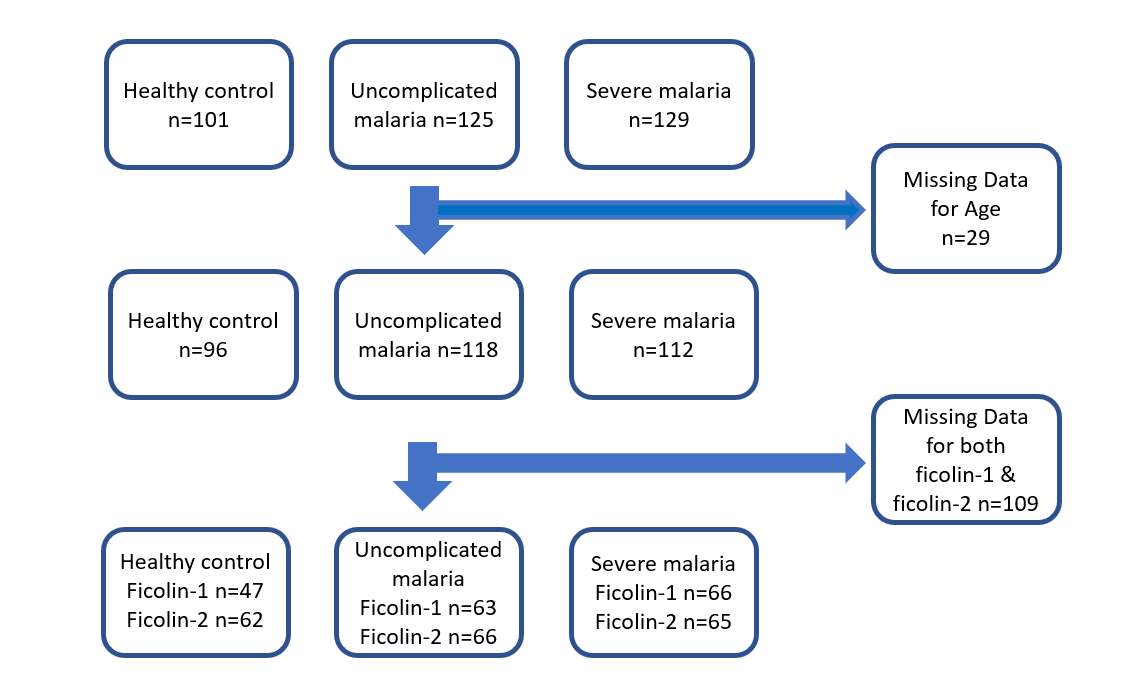


**Supplementary Figure 1**: Schematic of sample acquisition and measurement for ficolin-1 and ficolin-2.


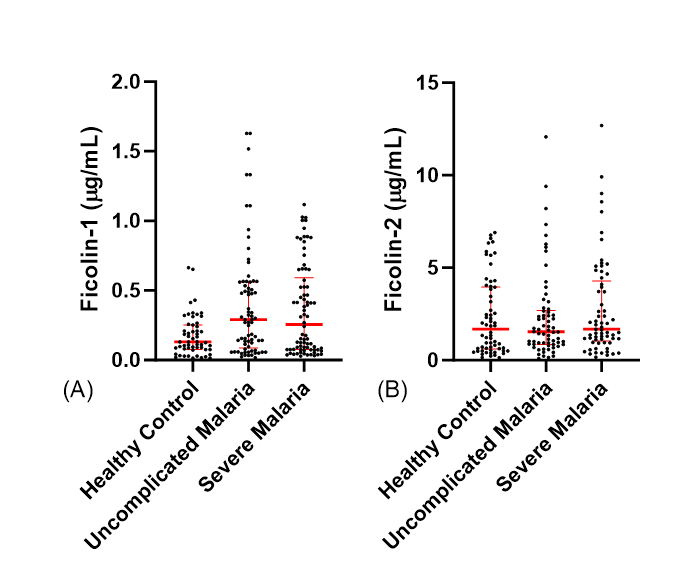


**Supplementary Figure 2:** Ficolin-1 (A) and ficolin-2 (B) levels in healthy controls, uncomplicated malaria and severe malaria. Scatterplot, lines median and interquartile range.
